# Antibiotic Resistance Genes and Associated Phenotypes in *Escherichia coli* and *Enterococcus* from Cattle at Different Production Stages on a Dairy Farm in Central California

**DOI:** 10.3390/antibiotics10091042

**Published:** 2021-08-26

**Authors:** Saharuetai Jeamsripong, Xunde Li, Sharif S. Aly, Zhengchang Su, Richard V. Pereira, Edward R. Atwill

**Affiliations:** 1 Research Unit in Microbial Food Safety and Antimicrobial Resistance, Department of Veterinary Public Health, Faculty of Veterinary Science, Chulalongkorn University, Bangkok 10330, Thailand; saharuetai.j@chula.ac.th; 2 Department of Population Health and Reproduction, University of California, Davis, CA 95616, USA; saly@ucdavis.edu (S.S.A.); rvpereira@ucdavis.edu (R.V.P.); 3 Veterinary Medicine Teaching and Research Center, School of Veterinary Medicine, University of California, Davis, CA 93274, USA; 4 Department of Bioinformatics and Genomics, University of North Carolina at Charlotte, Charlotte, NC 28223, USA; zcsu@uncc.edu

**Keywords:** dairy cattle, *Enterococcus*, *E. coli*, antibiotic resistance, gene

## Abstract

The objectives of this study were to characterize overall genomic antibiotic resistance profiles of fecal *Escherichia coli* and *Enterococcus* spp. from dairy cattle at different production stages using whole-genome sequencing and to determine the association between antimicrobial resistance (AMR) phenotypes and their corresponding genotypes. The Comprehensive Antibiotic Resistance Database (CARD) and ResFinder, two publicly available databases of antimicrobial resistance genes, were used to annotate isolates. Based on the ResFinder database, 27.5% and 20.0% of tested *E. coli* isolates (*n* = 40) harbored single and ≥3 antimicrobial resistance genes, respectively; for *Enterococcus* spp., we observed 87.8% and 8.2%, respectively. The highest prevalence of AMR genes in *E. coli* was for resistance to tetracycline (27.5%), followed by sulphonamide (22.5%) and aminoglycoside (20.0%); the predominant antimicrobial resistance genes in *Enterococcus* spp. targeted macrolide drugs (77.6%). Based on the CARD database, resistance to ≥3 antimicrobial classes was observed in all *E. coli* and 77.6% in *Enterococcus* spp. isolates. A high degree of agreement existed between the resistance phenotype and the presence of resistance genes for various antimicrobial classes for *E. coli* but much less so for isolates of *Enterococcus*. Consistent with prior work, fecal *E. coli* and *Enterococcus* spp. isolates from calves harbored a wide spectrum of resistance genes, compared to those from cattle at other production stages, based on the cross-sectional samples from the studied farm.

## 1. Introduction

Antimicrobial resistance (AMR) is a growing concern for food safety and public health globally [1]. Both humans and animals share similar antimicrobial drugs; hence, the judicious use of antimicrobials by both veterinary and human medicine is important to reduce the risk of AMR in enteric bacteria [2]. The administration of therapeutic and prophylactic antimicrobial drugs in animals can be at the individual animal or at the group (pen) level. Improper or excessive use of antimicrobials can lead to the development of AMR and multidrug resistance (MDR) in dairy cows [3] and calves [4,5,6], which could potentially result in the accumulation of bacterial AMR genes within livestock and throughout the farm environment.

Modern dairy production systems can be composed of multiple inter-connected cattle production stages, with each stage characterized by unique management practices [7]. Production status, disease conditions, and health status within the cattle groups, and patterns of and governing regulations for antimicrobial usage vary with these stages of production. The distribution of AMR genes in dairy farm settings has not been fully characterized due to the complexity of resistome in dairy production systems and different bacterial communities for different stages of production throughout the farm environment [8]. According to USDA’s Animal and Plant Health Inspection Service, antimicrobial use in dairy cattle production is classified as (1) three stages of dairy production consisting of preweaned heifers, weaned heifers, and cows and (2) treatment of digestive problems, respiratory infections, mastitis, lameness, and reproductive problems [9]. In general, the most frequent antimicrobials used in dairy cattle are tetracyclines, beta-lactams, cephalosporins, and florfenicols. Excessive selective pressures with high antimicrobial concentrations of relevant enteric bacteria can result in a high probability for selection, survival, and dissemination of AMR genes in the environment [3,10]. Although AMR genes are frequently detected in bacteria from dairy cattle feces [11], far less is known about the relative abundance of resistance in cattle at different production stages. These knowledge gaps of the ecological connectivity of AMR reservoirs in relation to their microbial communities, and AMR gene transmission pathways within and between dairy cattle at different production stages hamper our efforts to minimize the emergence and persistence of AMR. Whole-genome sequencing (WGS) and bioinformatics approaches are increasingly used to systemically characterize AMR genes in bacteria from livestock including dairy cattle [12,13,14,15].

The State of California has been the primary dairy producer in the US since 1993, contributing to 18.5% of US milk production [16]. In 2017, dairy cows in California accounted for greater than 20% of the entire dairy population in the US [17]. The overarching goal of this study was to characterize AMR genes in commensal bacteria from cattle at different production stages to generate data that can support future efforts to target AMR control efforts on the farms. Our objectives were to identify AMR genes in *Escherichia coli* (*E. coli*) (Gram negative) and *Enterococcus* spp. (Gram positive) from cattle at different production stages, contrast AMR phenotypes with the presence or absence of these bacterial AMR genes and identify production stages that have higher risks of spreading AMR genes within the farm environment.

## 2. Results

### 2.1. AMR Genes Detected in E. coli and Enterococcus spp.

**Figure 1:**
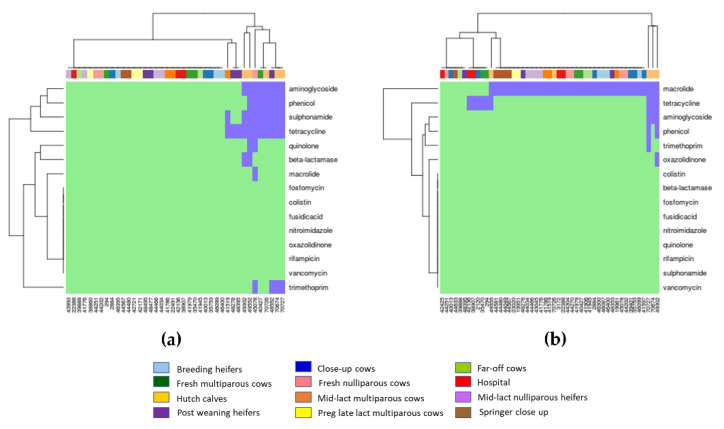
Heat map of resistance genes in *E. coli* and *Enterococcus* from dairy cattle at different production stages based on the detection (light blue color in the plot area) and non-detection of (light green color in the plot area) of resistance genes from the ResFinder database. Vertical axis is antimicrobial classes, and horizontal axis is cattle IDs: (**a**) *E. coli*; (**b**) *Enterococcus*.

**Figure 2:**
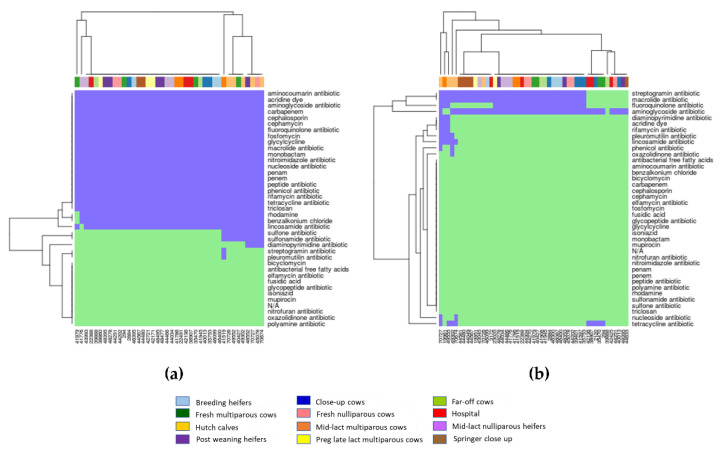
Heat map of resistance genes in *E. coli* and *Enterococcus* from dairy cattle at different production stages based on the detection (light blue color in the plot area) and non-detection (light green color in the plot area) of resistance genes from the CARD database. Vertical axis is antimicrobial classes, and horizontal axis is cattle IDs: (**a**) *E. coli*; (**b**) *Enterococcus*.

AMR genes detected in each of the 40 *E. coli* isolates and 49 *Enterococcus* spp. isolates conferring resistance to different antimicrobial drug classes are illustrated in heatmaps based on the detection of genes from the ResFinder and CARD databases in Figure 1 and Figure 2, respectively. Based on the detection of genes from the ResFinder database, 72.5% (29/40) of *E. coli* isolates were susceptible to all antimicrobials classes given they had no detectable AMR genes. The prevalence of *E. coli* isolates with at least one resistance gene to any of the antimicrobial classes was 27.5% (11/40), whereby 20.0% (8/40) of isolates contained ≥3 genes to different antimicrobial classes (Figure 1a). Five out of the eight *E. coli* isolates (62.5%) that contained genes to ≥3 different antimicrobial classes were from hutch calves, a substantially higher amount than expected by random chance given there are 12 different animal management categories with one being hutch calves (i.e., expect only ~0.7 MDR isolate per animal management category (8 isolates × (1/12) = 0.7 isolates per group)). The most abundant AMR genes identified in *E. coli* confer resistance to tetracycline (27.5%), followed by sulphonamide (22.5%), aminoglycoside (20.0%), and phenicol (20.0%). For *Enterococcus* spp., 87.8% (43/49) isolates had at least one resistance gene to any of the antimicrobial classes, and 8.2% (4/49) of isolates contained ≥3 genes to different antimicrobial classes (Figure 1b). Three out of the four MDR *Enterococcus* spp. isolates were from hutch calves, again, substantially higher than one would expect from random chance. The most common AMR genes detected in *Enterococcus* spp. were those conferring resistance to macrolide (77.6%).

Based on the detection of genes from the CARD database, all 40 *E. coli* isolates from cattle at the twelve different production stages had resistance genes to more than fifteen classes of antimicrobials (e.g., aminocoumarin, aminoglycoside, carbapenem, cephamycin, fluoroquinolone, glycylcycline, macrolide, monobactam, nucleoside, penams, peptide antibiotic, phenicol, rifamycin, tetracycline, acridine dye, fosfomycin, and nitroimidazole) (Figure 2a). For *Enterococcus*, 77.6% of isolates contained resistance genes to three classes of antimicrobials (Figure 2b). The most abundant genes detected in *Enterococcus* isolates conferred resistance to aminoglycoside (93.9%), macrolide (77.6%), and streptogramin (77.6%).

### 2.2. Associations between AMR Genes, Bacterial Species, and Production Stages

Logistic regression analysis was performed on all genes for *E. coli* and *Enterococcus* spp. detected from the two databases. Results indicated that the presence of macrolide resistance genes was negatively associated with tetracycline resistance genes in *Enterococcus* spp. (Table 1) based on the ResFinder database. There were no significant associations between other genes in *Enterococcus* spp. and genes in *E. coli* in the ResFinder and any genes in the two bacteria in the CARD database.

Due to the limited numbers of resistance genes detected in the ResFinder database for both *E. coli* and *Enterococcus* spp., statistical analyses of the presence of resistance and type of bacteria and production stage were performed only on genes detected from the CARD database. No significant association between the presence of resistance genes and type of bacteria and cattle production stages was observed (*p* > 0.05) among the 40 isolates of *E. coli*, but the small number of samples per production group (*n* = 3–5 isolates per group) may have limited the power of this analysis for the variable “cattle production stage”. However, macrolide resistance genes were more likely to be present in *Enterococcus* spp. than *E. coli* (*p* < 0.0001, OR = 134.7, and C.I. 16.6 to 1095.0) and fluoroquinolone resistance genes were negatively related with tetracycline resistance genes in *Enterococcus* spp. (Table 1).

Additional univariate logistic regression analyses comparing the prevalence of the each AMR gene among hutch calves, compared to the AMR prevalence in isolates from cattle at all other production stages, found that *E. coli* from hutch calves were much more likely to contain trimethoprim (OR = 22.7, *p* = 0.02) and phenicol (OR = 42.7, *p* = 0.003) resistance genes, while *Enterococcus* from hutch calves, compared to other cattle production stages, were much more likely to contain the tetracycline resistance genes (OR = 31.2, *p* = 0.005) based on ResFinder. For genes detected in the CARD database, *E. coli* from hutch calves, compared to bacteria from other cattle production stages, were more likely to contain the diaminopyrimidine antibiotic resistance genes (OR = 11.0, *p* = 0.040). Moreover, *Enterococcus* from hutch calves, compared to other cattle production stages, were more likely to contain diaminopyrimidine (OR = 29.3, *p* = 0.01), acridine dye (OR = 29.33, *p* = 0.01), rifampicin (OR = 14.3, *p* = 0.02), pleuromutilin (OR = 66.0, *p* = 0.002), lincosamide (OR = 86.0, *p* = 0.001), and tetracycline (OR = 32.0, *p* = 0.004) resistance genes.

### 2.3. Associations between AMR Genotypes and Phenotypes

Kappa analysis of the agreement between antimicrobial resistance genotypes and their phenotypes for all isolates is shown in Table 2. The results indicated significant agreement (range: 85.71–97.50%) between resistant phenotypes and the presence of resistance genes for *E. coli* identified by both the ResFinder (tetracycline, sulphonamide, trimethoprim, beta-lactamase, and aminoglycoside) and the CARD (tetracycline and aminoglycoside) databases. However, for *Enterococcus* spp., only tetracycline-resistant genes found in the ResFinder database were significantly associated with resistance phenotypes (85.7%).

### 2.4. Phylogenetic Characterization of AMR Gens in Dairy Cattle Production Line

**Figure 3:**
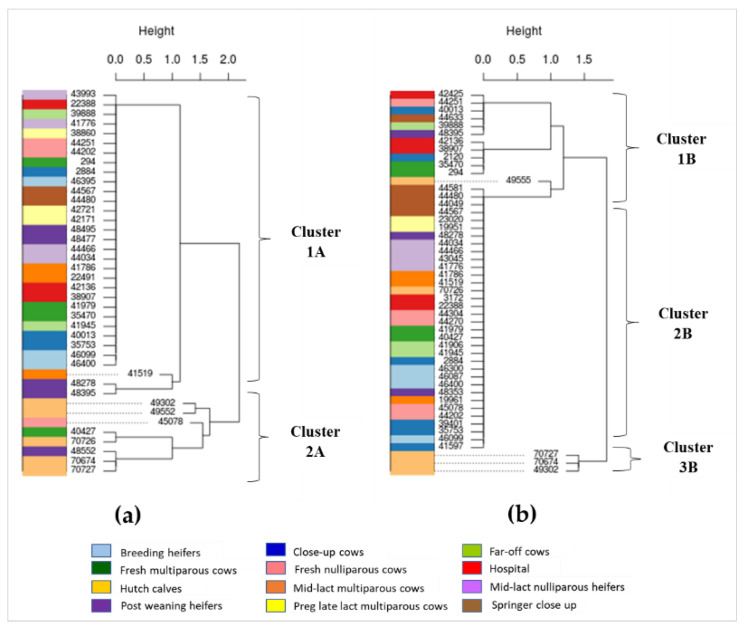
Phylogenetic clusters of *E. coli* and *Enterococcus* from dairy cattle at different production stages based on patterns of resistance genes detected from the ResFinder database. Numbers in the phylogenetic tree represent cattle IDs from which *E. coli* and *Enterococcus* were isolated: (**a**) *E. coli*; (**b**) *Enterococcus*.

**Figure 4:**
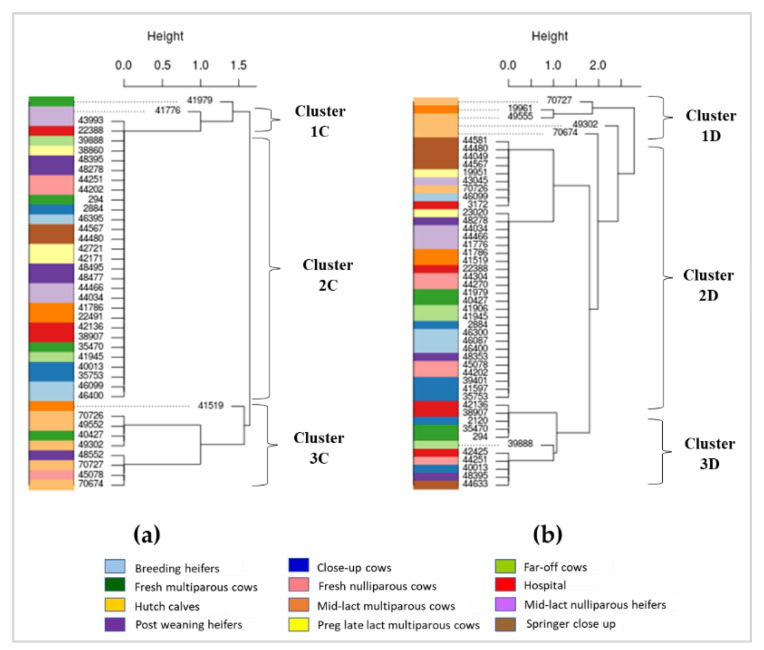
Phylogenetics of *E. coli* and *Enterococcus* from dairy cattle at different production stages based on resistance genes detected from the CARD database. Numbers in the phylogenetic tree represent cattle IDs from which *E. coli* and *Enterococcus* were isolated: (**a***) E. coli*; (**b**) *Enterococcus*.

Phylogenic trees were built separately for *E. coli* and *Enterococcus* spp. isolates from cattle at different production stages based on genes detected in the ResFinder database (Figure 3) and the CARD database (Figure 4). The phylogenetic tree of *E. coli* based on the ResFinder database indicated two distinct clades—namely, clusters 1A and 2A (Figure 3a).

Most *E. coli* isolates (*n* = 32/40) were in cluster 1A, which were susceptible to all antimicrobial classes. The cluster 2A was composed of *E. coli* isolates mostly from hutch calves (*n* = 5/8), which harbored resistance genes conferring aminoglycoside, sulphonamide, and tetracycline (Figure 1a and Figure 3a). *Enterococcus* spp. isolates were classified as clusters 1B, 2B, and 3B (Figure 3b). For cluster 1B (*n* = 12), half of (*n* = 6/12) *Enterococcus* spp. isolates had tetracycline-resistant genes, and hospital and fresh cows were the main sources of tetracycline-resistant isolates. Cluster 2B contained the majority of *Enterococcus* spp. isolates (*n* = 34) that were resistant to macrolide. Cluster 3B (*n* = 3) was composed of *Enterococcus* spp. isolates from all of the hutch calves, which harbored MDR genes conferring aminoglycoside, macrolide, and tetracycline (Figure 1b and Figure 3b).

Using genes detected from the CARD database, three clusters (1C, 2C, 3C) were identified among *E. coli* isolates (Figure 4a). All *E. coli* isolates in clusters 1C (*n* = 2), 2C (*n* = 29) and 3C (*n* = 9) contained resistance genes to numerous antimicrobial classes including acridine dye, aminocoumarin, aminoglycoside, benzalkonium chloride, carbapenem, cephalosporin, fluoroquinolone, fosfomycin, glycylcycline, macrolide, monobactam, nitroimidazole, nucleoside, penams, peptide antibiotic, phenicol, pleuromutilin, rifamycin, tetracycline, and triclosan. Isolates in cluster 1C were also resistant to antibacterial free fatty acids and cephamycin, whereas isolates in cluster 3C were resistant to lincosamide, rhodamine, sulfonamide, and sulfone. Hutch calves were the main source of isolates in cluster 3C (*n* = 5/9). Cluster 2C (*n* = 29) is the largest cluster of *E. coli* isolates that were obtained from all stages of dairy cattle production; almost all these isolates except one shared the exact same pattern of multiple antimicrobial resistance genes (Figure 2a (i.e., large block of blue highlight) and Figure 4a). *Enterococcus* spp. isolates were categorized into three clusters (1D, 2D, and 3D) based on the detection of genes in the CARD database (Figure 4b). *Enterococcus* spp. isolates in cluster 1D (*n* = 5) were mostly from hutch calves (*n* = 4) and isolates in this cluster were resistant to lincosamide, macrolide, streptogramin followed by phenicol and tetracycline. *Enterococcus* spp. isolates in cluster 2D (*n* = 33) were distributed across all stages of dairy production. All isolates in this cluster were resistant to streptogramin, macrolide, and aminoglycoside, and 94% (31/33) of these isolates were also resistant to fluoroquinolone. *Enterococcus* spp. isolates in cluster 3D (*n* = 11) were susceptible to most antimicrobial classes except for aminoglycoside (*n* = 10) and tetracycline (*n* = 5), and most of the resistant isolates were from hospital (*n* = 3) and close up (*n* = 2) dairy cows (Figure 2b and Figure 4b).

## 3. Discussion

### 3.1. Abundance of AMR Genes in E. coli and Enterococcus spp.

The purpose of our study was in part to characterize the overall resistance profile of fecal *E. coli* and *Enterococcus* from cattle at different production stages. Based on the resistance genes detected from the ResFinder database (Figure 1), genes conferring resistance to tetracycline, sulphonamide, and aminoglycoside were the main resistance genes in *E. coli*. This finding was similar to a previous study of AMR in *E. coli* isolated from dairy cattle, which found *E. coli* was mostly resistant to tetracycline (93%) followed by florfenicol (78%), ampicillin (48%), and chloramphenicol (20%) [18]. For *Enterococcus* spp., resistance to macrolide was the main resistance gene identified in the ResFinder database (Figure 1). In terms of resistance genes identified from the CARD database, 100% of *E. coli* isolates had genes resistant to over 15 classes of antimicrobials, and 77.6% of *Enterococcus* isolates had genes resistant to three classes of antimicrobials. Due to the differences in availability and settings of genes between the ResFinder and CARD databases, it was not surprising that resistance genes in *E. coli* and *Enterococcus* identified from the two databases were not identical. Interesting, the two databases were consistent in the detection of tetracycline, aminoglycoside, and phenicol as major resistant genes in *E. coli* and macrolide and aminoglycoside as major resistant genes in *Enterococcus*.

With respect to the major resistance in *E. coli* (i.e., tetracycline) and *Enterococcus* (i.e., macrolide), tetracycline is one of the commonly used antimicrobials in food animal production in the US and Europe, frequently for digestive conditions [19]. Tetracycline is normally used for the treatment of respiratory diseases in food-producing animals in the US [20]. Tetracycline-resistant bacteria, especially non-pathogenic or commensal bacteria, may play a major role as bacterial reservoirs for AMR and MDR, both within cattle populations and for the general dairy farm environment given the ubiquity of manure in these production systems. In general, macrolides and lincosamides are used for the treatment of bacterial infection, especially in mastitis cows, and for growth promotion in food-producing animals. Macrolides are also used in combination with aminoglycosides to treat mastitis in dairy cattle in some European countries, while lincosamides are mainly used in the US in dairy cattle production [21]. We did not collect information on antimicrobial use for this study; hence, we were unable to assess the relationships between the occurrence of AMR genes and antimicrobial use on the farm. However, many studies have indicated that the use of antimicrobials in food-producing animals including dairy cows can lead to increases in AMR and MDR bacteria on livestock farms [19,21]. In future studies, it would be interesting to further investigate the relationships between AMR and patterns of antimicrobial use at different production stages.

### 3.2. Associations between AMR Genes, Bacteria, and Production Stages

*Enterococcus* spp. are known to cause mastitis in dairy cattle [22]. A previous study revealed that *Enterococcus* spp. isolates from fecal samples from 122 dairy cattle operations were resistant to lincomycin (92.3%), followed by flavomycin (71.9%), and tetracycline (24.5%) [21]. In the current study, the majority of *Enterococcus* spp. isolates that were susceptible to macrolides were found in hospital cows, similar to previous work demonstrating that *Enterococcus* resistance to macrolides was found in isolates from clinical animals [23]. Moreover, all MDR *Enterococcus* isolates were from hospital and fresh cows, indicating that macrolide resistance genes might originate from hospital cows that are being treated for a variety of medical conditions and then spread to fresh cows.

Among *Enterococcus* isolates, a negative association was noted between the occurrence of tetracycline and macrolide genes, indicating that the presence of the tetracycline resistance genes was associated with a reduced risk of simultaneously finding the macrolide resistance gene in these fecal bacteria. This finding is interesting given the previous observation that resistance to tetracycline and macrolide–lincosamide–streptogramin (MLS) group was observed through transposable elements [24]. In dairy production, lincosamide is used to treat mastitis in conventional farms; however, lincosamide resistance genes were found in hutch calves based on the CARD database. This may indicate the transfer of resistance genes along the production line and calves can acquire resistance genes at this early age. Similarly, it was reported that calves at 1–2 weeks of age acquired tetracycline-resistant genes, likely due to colonization with resistant bacteria from their mothers and/or the dairy farm environment, given the ubiquity of manure [25]. According to genes identified from the CARD database, resistance to ≥3 antimicrobial classes genes was commonly observed among *E. coli* and *Enterococcus* spp. No significant links between resistance to tetracycline and fluoroquinolone were observed in this study, which may be due to the mechanism of resistance to fluoroquinolone being frequently related to chromosomal mutations, while the mechanism of resistance to tetracycline can occur due to genetic mobility [26].

### 3.3. Correlations between AMR Genotypes and Phenotypes

For this study, we identified genes by evaluating two publicly available databases in *E. coli*—namely, sulphonamide, trimethoprim, and beta-lactamase resistance genes from ResFinder, and tetracycline and aminoglycoside resistance genes from both ResFinder and CARD. These resistance genotypes were in concordance with resistance phenotypes we characterized previously [6]. For *Enterococcus* spp., high levels of agreement between resistance genotypes and phenotypes [6] were only found for tetracycline resistance genes from the ResFinder (Table 2). Similarly, a previous study observed a lower concordance between phenotypes and genotypes of streptomycin (specificity as low as 5.6%) in *Salmonella* isolates [27]. In contrast, high correlations (99.0%) between the presence of resistance genotypes and observed phenotypes have been reported in nontyphoidal *Salmonella* from retail meat specimens and human cases [28]. Another study reported 67.9–100% concordance between resistance phenotypes and genotypes and 98.0–99.6% concordance between susceptible phenotypes and genotypes in *Campylobacter* from retail poultry [29]. Although relatively few studies have been performed on Gram-positive organisms using WGS to study AMR, a high correlation (96.5%) between resistance genotypes and phenotypes in *Enterococcus* isolates was reported [30]. The lower correlations between resistance genotypes and phenotypes of *Enterococcus* in the current study could be due to the small numbers of bacterial isolates tested, availability of drugs for antimicrobial susceptibility test in the commercial kits, and a different method used to analyze correlations between genotypes and phenotypes. In addition, the lower correlations also could be due to discrepancies between genotype and phenotype resistance that vary with bacterial species and antimicrobials [31,32,33,34]. Therefore, a combination of genotypes for resistance prediction with phenotypes determined by antimicrobial susceptibility would provide a more accurate assessment of resistance of different bacterial species from different samples and against different antimicrobials. Results of genotypes in the current work and phenotypes in our previous work [6] on the same bacterial strains allowed us to better understand the resistance of *E. coli* and *Enterococcus* spp. on dairy farms.

### 3.4. Phylogenetics of AMR Genes in Cattle at Different Production Stages

Based on phylogenetic analysis of resistance genes in *E. coli* detected from the ResFinder database, a quarter of the isolates that were in cluster 2A (Figure 3a) were from hutch calves. Phylogenetic analysis of resistance genes of *Enterococcus* detected from ResFinder also indicated a unique cluster of MDR genes mainly from hutch calves (i.e., cluster 3B in Figure 3b). Similarly, phylogenetic analysis of genes detected from the CARD database found distinct clusters of genes in *E. coli* (cluster 3C in Figure 4a) and *Enterococcus* (cluster 1D in Figure 4b) from hutch calves. Therefore, these results indicate that bacteria from hutch calves had AMR characteristics that were distinct from isolates from cattle at other stages of dairy production. Most *E. coli* isolates from hutch calves were MDR to aminoglycoside, phenicol, sulphonamide, and tetracycline, which is consistent with other studies in that *E. coli* from calves were frequently resistant to multiple antimicrobials. For example, MDR bacteria were very common from integrated veal calves [4]. A review article indicated that young dairy calves often carry high levels of AMR in their fecal *E. coli* and *Salmonella enterica*, which could provide a potential reservoir of AMR genes for the greater dairy farm environment depending on how calf manure is managed or mixed into the general manure stream on the dairy [5]. Our results, in addition to these prior studies and reviews, suggest that monitoring of MDR bacteria in hutch calves may be important for reducing the spread of AMR bacterial genes to other production stages in dairy farm settings. On the other hand, heat maps and phylogenetic analyses indicated a wide distribution of multiple resistance genes among multiple adult cattle production stages for fecal *E. coli* based on the CARD database (e.g., the large blue block in Figure 2a). Given that one adult dairy cow can produce in excess of 20 to 30 kg of feces a day, conventional dairy herd sizes in California often exceed 1000 adult cows, and the concentration of fecal *E. coli* in dairy manure typically exceeds 10^6^ cfu/g (10^9^ cfu/kg), one can expect that MDR fecal bacteria are widely distributed throughout the greater dairy farm environment and likely in relatively high concentrations. A previous study reported that AMR gene profiles varied between farms and different types of samples (fecal, manure, and soil) but a greater proportion of genes were common to all types of samples, suggesting horizontal transfer of common resistance genes among production stages [35]. Samples in this study were collected within one farm at one point in time, and the sample size from each production stage was small due to the cost of WGS and available funding; these constraints may limit the representativeness of our results. However, our study warrants further investigation of the relationship between AMR clusters in different cattle groups and different types of farm sample matrices (e.g., manure, soil) to support the effort to better control the spread of AMR within modern conventional dairy farms.

## 4. Materials and Methods

### 4.1. Bacterial Isolates and Resistance Phenotypes

In our previous work, we characterized the antimicrobial resistance phenotypes of *E. coli* and *Enterococcus* spp. from cattle at different production stages on a commercial dairy farm in Central California, USA [6]. Briefly, using convenient sampling, fecal samples were collected from the rectum of dairy cattle at twelve different production stages on a commercial farm in the San Joaquin Valley, the major dairy production region of California. The antimicrobial susceptibility of *E. coli* and *Enterococcus* strains was determined by minimum inhibition concentrations (MIC) of tested antimicrobials using a microbroth dilution method [6]. Antimicrobials tested for *E. coli* were cefoxitin, azithromycin, chloramphenicol, tetracycline, ceftriaxone, amoxicillin/clavulanic acid, ciprofloxacin, gentamycin, nalidixic acid, ceftiofur, sulfisoxazole, trimethoprim–sulfamethoxazole, ampicillin, and streptomycin. Antimicrobials tested for *Enterococcus* were tigecycline, tetracycline, chloramphenicol, daptomycin, streptomycin, tylosin tartrate, quinupristin/dalfopristin, linezolid, nitrofurantoin, penicillin, kanamycin, erythromycin, ciprofloxacin, vancomycin, lincomycin, and gentamycin [6]. Resistance phenotypes (i.e., resistant or susceptible to tested antimicrobials) of *E. coli* and *Enterococcus* from the previous work were used for the analysis of associations with genotypes in the current work.

In the current study, based on the availability of strains from cattle at different production stages that determined resistance phenotypes in our previous work, 40 strains of *E. coli* and 49 strains of *Enterococcus* from our culture collections were selected for genotype characterization using whole-genome sequencing (Table 3).

### 4.2. DNA Sequencing and Assembly

Bacterial isolates were inoculated to 200 μL of LB broth (Difco Laboratories, Plymouth, MI, USA) and incubated overnight at 37 °C for *E. coli* and at 41 °C for *Enterococcus* spp. Bacterial cells were centrifuged at 10,000 rpm for 10 min. Genomic DNA was extracted from pelleted cells using the PowerMax Soil DNA Isolation kit (QIAGEN company, Venlo, The Netherlands) according to the manufacturer’s instructions. Barcode-indexed sequencing libraries were generated from 300 ng genomic DNA for each bacterial isolate using the Illumina Nextera DNA Flex Library kit (Illumina, San Diego, CA, USA) following the manufacturer’s instructions. The sequencing libraries were amplified with five PCR cycles. The fragment lengths distribution of the sequencing libraries was analyzed with a Bioanalyzer 2100 instrument (Agilent, Santa Clara, CA, USA). The libraries were quantified by fluorometry on a Qubit instrument (Life Technologies, Carlsbad, CA, USA), and combined equimolarly into a single pool. Then, the pool was quantified by qPCR with a Kapa Library Quant kit (Kapa Biosystems-Roche, Wilmington, MA, USA) and sequenced on one lane of an Illumina HiSeq 4000 (Illumina, San Diego, CA, USA) with paired-end 150 bp reads. The sequencing was carried out at the DNA Technologies and Expression Analysis Core Laboratory at the UC Davis Genome Center. Read pairs for each sample were preprocessed using htstream-1.0.0 (https://github.com/ibest/HTStream, last accessed on 24 January 2019), collecting basic read stats and quality information, screening to remove phiX sequence, and trimming Illumina adapters. Each sample’s read set was assembled de novo using SPAdes 3.12.0 [36].

### 4.3. Bioinformatics Analysis

For gene annotation of assembled genomes, the genes encoded in the genome and plasmids were identified using the GeneMark14 and Glimmer15 software, and both were hidden Markov model-based gene-finding tools. Potential AMR genes in each assembled genome were identified by finding homologs of the predicted genes in the publicly available resistance genes database of the ResFinder (https://cge.cbs.dtu.dk/services/ResFinder/, last accessed on 24 January 2019) [37] and the Comprehensive Antibiotic Resistance Database (CARD) (https://card.mcmaster.ca/, last accessed on 24 January 2019) [38]. AMR gene sequences downloaded from CARD and ResFinder databases were aligned to the assembled scaffolds for each sample using BWA MEM [39]. Each gene was counted as present in an assembly if an alignment covering 90% of the gene’s length was detected, using a custom python script. Genes not detected in any samples were discarded and then gene counts were clustered by gene, and by sample using R’s “hclust” function. To characterize the overall resistance profile of fecal bacteria from different production stages, homologs of genes were compiled and expressed as antimicrobial classes a bacterial isolate resistant to, instead of specific genes detected.

According to the identification of horizontal gene transfer (HGT), patterns of the AMR genes among resistant isolates, phylogenetic analyses were conducted based on the HGT pattern of AMR genes in resistant bacteria from different production stages. The amino acid sequences for unique AMR genes in different isolates were aligned using ClustalX with the GONNET protein weight matrix. Unrooted phylogenetic trees were generated from ClustalX alignments, using the neighbor-joining algorithm based on the principle of minimum evolution, along with bootstrap analysis with 1000 replicates. The trees were plotted using DRAWTREE in the PHYLIP package. The HGT events were identified by comparison of the AMR gene trees of resistant isolates using the parsimonious principle.

### 4.4. Statistical Analyses

Descriptive statistics were used to examine the distribution of AMR and MDR genes in *E. coli* and *Enterococcus* detected from the CARD and ResFinder databases. Logistic regression analysis was used to identify the relationship between the presence of various resistance genes. The production stages and bacterial species were also included as independent variables for the regression models. Univariate regression analysis for all independent variables was screened for potential significance, and a *p*-value threshold of 0.05 was used as an inclusion criterion in the model. Backward and forward stepping algorithms were used to identify multivariate regression models.

Kappa coefficient analysis was used to assess the level of agreement (interrater reliability) between a bacterial isolate having a specific resistance genotype and also having the corresponding resistant phenotype for the isolates. The resistance genotypes used in the Kappa analysis were the genes detected from the CARD and ResFinder databases, while the resistance phenotypes were determined from our work on the same set of samples published previously [6]. For the Kappa analysis, each bacterial isolates’ phenotypic resistance was compared to its corresponding pattern of resistance genes (presence/absence) by classes of antimicrobial drugs. The percentage generated by the Kappa analysis indicated the degree of agreement between a pair of resistance phenotype and genotype class. A Kappa value of 100% indicated perfect agreement, while a Kappa 0% means no agreement between the presence of an AMR genotype class and its associated phenotype and. All statistical analyses were performed using Stata version 14 (StataCorp, College Station, TX, USA). A *p*-value < 0.05 was considered as statistical significance.

## 5. Conclusions

This study characterized the antimicrobial resistance profiles of fecal *E. coli* (Gram negative) and *Enterococcus* spp. (Gram positive) from cattle at different production stages on a commercial dairy farm and tested the association between resistance phenotypes and their corresponding genotype class. Results indicated that based on the CARD database of resistance genes, a large proportion of AMR genes were common to all dairy production stages for fecal *E. coli*, yet unique clusters of specific AMR genes existed in hutch calves for both *E. coli* and *Enterococcus* that may function as on-farm reservoirs of AMR and thereby promote the horizontal transfer of resistance genes to cattle in other production stages. Furthermore, a high degree of agreement existed between the resistance phenotype and the presence of resistance genes for various antimicrobial classes for *E. coli* but much less so for isolates of *Enterococcus.* With respect to this group of fecal *E. coli* in these dairy cattle, this high degree of agreement from the Kappa analysis suggests that if an AMR gene is present on these cattle fecal bacteria, the gene presumably is being expressed to the extent that the corresponding AMR phenotype is also evident based on the MIC values. A better understanding of the dynamic relationship between AMR phenotypes and their underlying genotypes and the mechanisms that would reduce AMR gene expression should contribute to the growing repertoire of strategies to reduce AMR in cattle on dairy production farms.

## Figures and Tables

**Table 1:** Logistic regression analysis between genotypic resistance and resistance genes of *Enterococcus* spp. (*n* = 49).

Source	Resistance Gene	Factor	Coeff.	Std. Err.	C.I.	*p*-Value
ResFinder	Macrolide	constant tetracycline	1.735 −1.958	0.443 0.804	0.867 to 2.602 −3.533 to −0.382	<0.0001 0.015
CARD	Fluoroquinolone	constant tetracycline	0.446 −1.700	0.320 0.863	−0.181 to 1.074 −3.391 to −0.007	0.163 0.049

Coeff: coefficient; C.I.: confidence interval; Std. Err.: standard error.

**Table 2:** Kappa analysis among phenotypic and genotypic characterization of resistance *E. coli* (*n* = 40) and *Enterococcus* spp. (*n* = 49).

Source	Genotype	Phenotype	Agreement (%)	Expected Agreement (%)	Kappa (%)	Std. Err.	*p*-Value
ResFinder ^a^	TET	TET	95.00	57.88	88.13	0.157	<0.0001
ResFinder ^a^	SUL	SXT	97.50	88.25	78.72	0.155	<0.0001
ResFinder ^a^	TRI	SXT	87.50	75.50	48.98	0.158	0.0002
ResFinder ^a^	BET	AMP	97.50	88.25	78.72	0.136	<0.0001
ResFinder ^a^	AMI	STR	95.00	71.00	82.76	0.155	<0.0001
CARD ^a^	TET	TET	85.71	63.56	60.80	0.156	<0.0001
CARD ^a^	AMI	STR	89.80	83.13	39.51	0.137	0.0015
ResFinder ^b^	TET	TET	85.71	63.56	60.80	0.133	<0.0001

^a^*E. coli*; ^b^*Enterococcus* spp.; Std. Err.: standard error. AMP: ampicillin; AMI: aminoglycoside; BET: beta-lactamase; STR: streptomycin; SUL: sulphonamide; SXT: trimethoprim–sulfamethoxazole; TET: tetracycline; TRI: trimethoprim.

**Table 3:** Number of *E. coli* and *Enterococcus* isolates from twelve different production stages used in genotype characterization in this study.

Management Units	*E. coli*	*Enterococcus*
Hutch calves	5	5
Post-weaning heifers	5	3
Breeding heifers	3	4
Springers/Close-up yearlings	2	5
Fresh uniparous cows	3	6
Fresh multiparous cows	4	3
Mid lactation uniparous cows	3	3
Mid-lactation multiparous cows	4	4
Pregnant late lactation multiparous cows	3	2
Far-off multiparous cows	2	3
Close up multiparous cows	3	6
Hospital animals	3	5
Total	40	49

## Data Availability

The publicly available resistance genes database of the ResFinder (https://cge.cbs.dtu.dk/services/ResFinder/ (last accessed on 24 January 2019)) and the Comprehensive Antibiotic Resistance Database (CARD) (https://card.mcmaster.ca/ (last accessed on 24 January 2019)) were used to identify resistance genes in *E. coli* and *Enterococcus*. The genomic data of *E. coli* and *Enterococcus* are available on request from the corresponding authors.
